# COT: an efficient and accurate method for detecting marker genes among many subtypes

**DOI:** 10.1093/bioadv/vbac037

**Published:** 2022-05-27

**Authors:** Yingzhou Lu, Chiung-Ting Wu, Sarah J Parker, Zuolin Cheng, Georgia Saylor, Jennifer E Van Eyk, Guoqiang Yu, Robert Clarke, David M Herrington, Yue Wang

**Affiliations:** 1 Department of Electrical and Computer Engineering, Virginia Polytechnic Institute and State University, Arlington, VA 22203, USA; 2 Advanced Clinical Biosystems Research Institute, Cedars Sinai Medical Center, Los Angeles, CA 90048, USA; 3 Department of Internal Medicine, Wake Forest University, Winston-Salem, NC 27157, USA; 4 The Hormel Institute, University of Minnesota, Austin, MN 55912, USA

## Abstract

**Motivation:**

Ideally, a molecularly distinct subtype would be composed of molecular features that are expressed uniquely in the subtype of interest but in no others—so-called marker genes (MGs). MG plays a critical role in the characterization, classification or deconvolution of tissue or cell subtypes. We and others have recognized that the test statistics used by most methods do not exactly satisfy the MG definition and often identify inaccurate MG.

**Results:**

We report an efficient and accurate data-driven method, formulated as a Cosine-based One-sample Test (COT) in scatter space, to detect MG among many subtypes using subtype expression profiles. Fundamentally different from existing approaches, the test statistic in COT precisely matches the mathematical definition of an ideal MG. We demonstrate the performance and utility of COT on both simulated and real gene expression and proteomics data. The open source Python/R tool will allow biologists to efficiently detect MG and perform a more comprehensive and unbiased molecular characterization of tissue or cell subtypes in many biomedical contexts. Nevertheless, COT complements not replaces existing methods.

**Availability and implementation:**

The Python COT software with a detailed user’s manual and a vignette are freely available at https://github.com/MintaYLu/COT.

**Supplementary information:**

Supplementary data are available at *Bioinformatics Advances* online.

## 1 Introduction

An important but frequently underappreciated issue is how best to define and detect a cell or tissue marker among many subtypes. Ideally, a molecularly distinct subtype would be composed of molecular features that are expressed uniquely in the cell or tissue subtype of interest but in no others—so-called marker genes (MGs; Kuhn *et al.*, 2011). With the increasing availability of subtype expression profiles acquired by single or sorted cell sequencing, data-driven software tools to detect MG are essential for characterization, classification or deconvolution of tissue or cell subtypes (Herrington *et al.*, 2018; Parker *et al.*, 2020).

The most frequently used methods rely on an ANOVA model that adopts the null hypothesis that samples in all subtypes are drawn from the same population and are originally designed to detect differentially expressed genes across any of the subtypes. Another popular method is the One-Versus-Rest Fold Change or *t*-test (OVR-FC/*t*-test/Limma/EdgeR) that is based on the ratio of the averaged expression in a particular subtype to the averaged expression in all other subtypes (Chikina *et al.*, 2015; Ritchie *et al.*, 2015). However, a gene with a low average expression value in the rest is not necessarily expressed at a low level in every subtype in the rest. An alternative strategy is the One-Versus-Everyone Fold Change (OVE-FC) or its variants (Newman et al., 2015; Chen *et al.*, 2021). Because an OVE test compares only the top two subtypes (with the highest or second-highest averaged expression value) for mathematical convenience, the remaining informative subtypes are neglected. We and others have recognized that these test statistics used by most current methods do not satisfy exactly the MG definition and are theoretically prone to detecting inaccurate MG (Kuhn *et al.*, 2011).

**Fig. 1.**
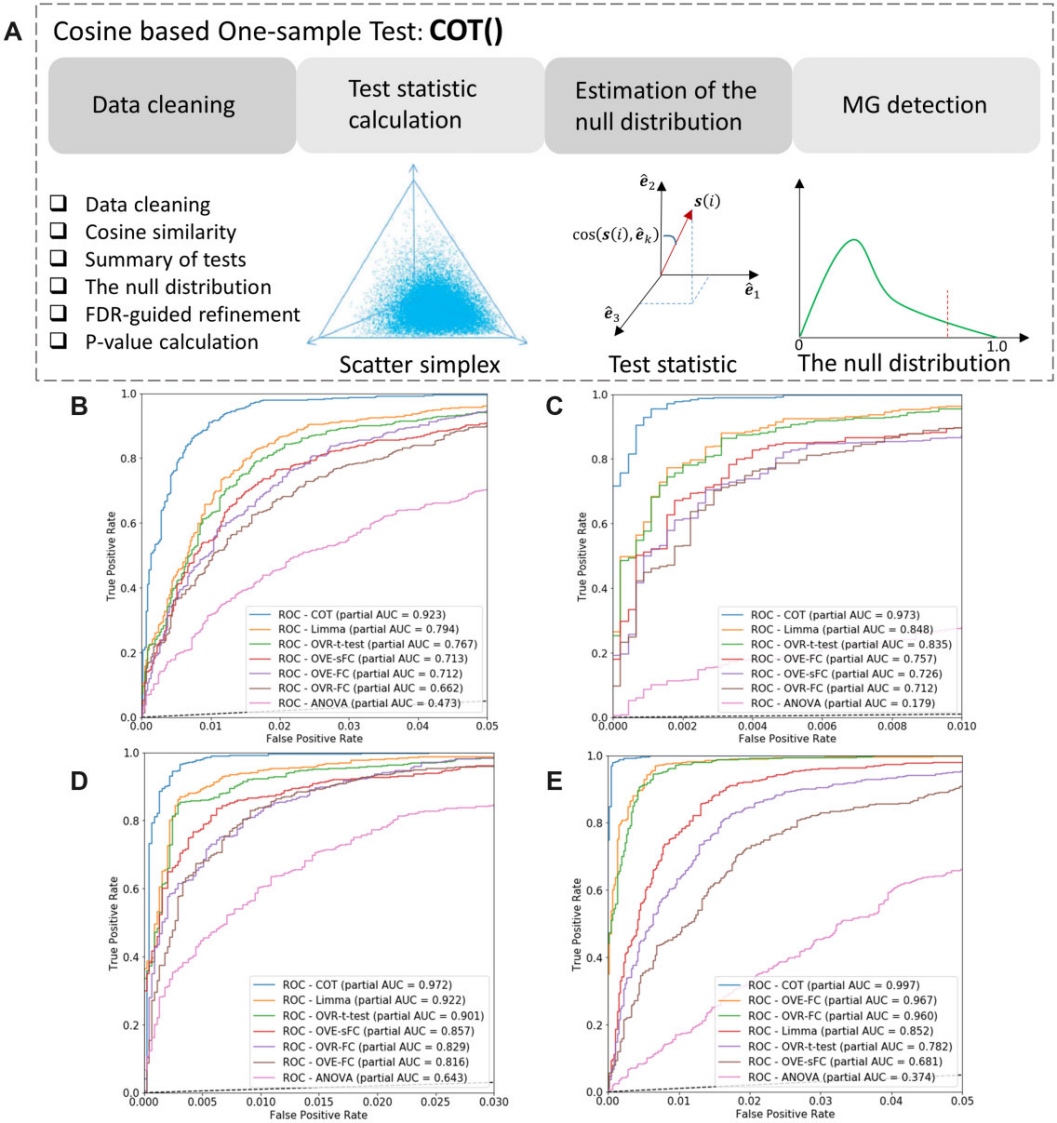
Overall COT workflow and comparative evaluation. (**A**) Major steps and functions in COT software tool. (**B–E**) pROC curves and pAUC values associated with COT and peer methods in the standard (*K *=* *3, *n *=* *3 × 3), more-subtype (*K *=* *5, *n *=* *3 × 5), more-sample (*K *=* *3, *n *=* *4 × 3) and complex-null (mixture of rotated and non-uniform Dirichlet distributions) experimental settings, respectively (Supplementary information)

Here, we report an accurate and efficient data-driven method—Cosine-based One-sample Test (COT)—to detect MG among many subtypes (Fig. 1A). Formulated as a one-sample test, the test statistic of COT is the cosine similarity between a molecule’s expression pattern across all subtypes and the exact mathematical definition of an ideal MG. Under the assumption that most genes are associated with the null hypothesis, COT approximates the empirical null distribution with a finite normal mixture (FNM) distribution for calculating *P*-values (Efron, 2004). We implement the COT workflow in a Python package, evaluate and compare MG detection by COT and peer methods using realistic simulation data. We demonstrate the superior performance and utility of COT on gene expression and proteomics data acquired from enriched tissue or cell subtype samples, where biomedical case studies have led to novel findings and hypotheses.

## 2 Methods

### 2.1 Problem formulation and test statistic

Mathematically, an ideal MG of subtype *k* is defined as a gene expressed only in subtype *k* but not in any other subtypes (Chikina *et al.*, 2015; Delaney *et al.*, 2019; Kuhn *et al.*, 2011), approximately

(1) skiMG, k≫0,sl≠kiMG, k≈0,#

where $s_{k}\left(i_{\text{MG},\;k}\right)$ and $s_{l\neq k}\left(i_{\text{MG},\;k}\right)$ are the average expressions of marker gene $i_{\text{MG},\;k}$ in subtypes *k* and l, respectively. We acknowledge that there are alternative definitions but in the absence of a universally accepted standard in the field, our definition provides some unique advantages to guide our work. We emphasize that subtype-specific MG as defined here are enriched uniquely in a particular subtype, regardless of their expression level(s), and their identities can be readily used in facilitating deconvolution or classification (mathematically proven Theorem 1; Wang *et al.*, 2016; Supplementary information).

Accordingly, the cross-subtype expression pattern of an ideal MG can be represented concisely by the Cartesian unit vectors ${\hat{e}}_{k}$, readily serving as a reference for a one-sample test. Conceptually, the null hypothesis for non-MG, and the alternative hypothesis for MG, can be described as

(2) Hnon-MGnull: si≠e^k;HMGalternative: si=e^k;#

where $s\left(i\right)=\left[s_{1}\left(i\right),\;s_{2}\left(i\right),\;\ldots ,\;s_{K}\left(i\right)\right]$ is the sample-averaged cross-subtype expression pattern of gene *i*. Fundamental to the success of COT is the newly proposed test statistic $\cos\left(s\left(i\right),\;{\hat{e}}_{k}\right)$ that measures directly the similarity between the cross-subtype expression pattern $s\left(i\right)$ of gene *i* and the ideal MG expression pattern of constituent subtypes in scatter space given by Figure 1A.

(3) $$\begin{array}{c} t_{\text{COT}}(i_{\text{MG}})=\underset{1\leq k\leq K}{\mathrm{argmax}}\cos\left(s\left(i\right),\;{\hat{e}}_{k}\right)=\underset{1\leq k\leq K}{\mathrm{argmax}}\frac{s_{k}\left(i\right)}{\sqrt{\sum_{j=1}^{K}{\left[s_{j}(i)\right]}^{2}}},\# \end{array}$$

where *K* is the number of constituent subtypes. Because $s\left(i\right)$ is confined within the first quadrant where the central vector is the ‘all-ones’ vector $\vec{1}$, we have $1/\sqrt{K}<t_{\mathrm{COT}}\left(i\right)<1$ (Fig. 1A).

### 2.2 COT workflow and software

Sample normalization and batch effect adjustment are the required preprocessing steps prior to COT analysis; when applicable, the input of COT should be a sample-normalized and batch-adjusted data matrix. The COT workflow consists of four major analytics steps (Fig. 1A, Supplementary method):

1. Data Cleaning. Molecule features whose expression levels across all subtypes are lower than a prefixed lower bound, or whose norms across all subtypes are higher than a prefixed higher-bound, are removed (noise or outlier).
2. Test Statistic Calculation. For each of the remaining genes, cosine similarity $\cos\left(s\left(i\right),\;{\hat{e}}_{k}\right)$ between the cross-subtype averaged expression pattern and the ideal MG reference is calculated.
3. Null Distribution Approximation. The empirical null distribution is summarized over all genes and may be approximated by an FNM distribution.
4. MG Detection. Based on the observed test statistic and null distribution, an MG is identified subject to a proper one-sided significance threshold.

We implemented the COT workflow in Python and used community-based trials to test the COT software. The Python package is open source at GitHub, built using NumPy and Pandas, and is distributed under the MIT license. The COT software tool is easy to use and applicable to multiomics data. The rows of input data matrix correspond to genes or other molecular features, and the columns correspond to samples. The subtype label on each sample is required by the COT test statistic. The output file stores the input genes and their cosine values in reference to the ideal MG of respective subtypes (Supplementary scripts).

### 2.3 Performance index

We use two qualitative and three quantitative criteria to assess the quality of MGs detected by COT or peer methods. The two qualitative measures are scatter simplex and MG heatmap. In the simulation studies, we use receiver operating characteristic (ROC) analysis to evaluate the accuracy of MG detection by COT and peer methods against the ground truth. For benchmark verification, we first propose a quantitative and more objective performance index, given by

(4) P1=1M(K-1)∑i=1M∑j=1KsjiMGmaxkskiMG-1,#

where M is the number of MGs, and $0\leq P_{1}\leq 1$, to evaluate the quality of MGs detected by COT and peer methods. By the definition of MG, the ideal MG matrix corresponds to a row-permutation matrix; on each column, only one of the elements is equal to unity while all the other elements are zero. Clearly, the index (4) attains its minimum value zero for an ideal set of MGs. The larger the value of $P_{1}$, the poorer the quality of MG candidates. We then propose MG-guided deconvolution accuracy (i.e. difference and/or correlation between true and estimated mixing proportions) to assess the quality of MGs detected by COT and peer methods (Chen *et al.*, 2020).

## 3 Results

We conducted three-phased experiments to evaluate the performance and report the utility of COT and its Python software tool, including comparisons between COT and peer methods using simulation data, verification of MG detected by COT on benchmark real gene expression data and a case study of COT application on real proteomics data.

### 3.1 Evaluation and comparison of COT and peers using simulation data

We conducted extensive experiments to evaluate the performance of COT and five peer methods using realistic simulation data. The peer methods in comparison include ANOVA, Limma/EdgeR, OVR *t*-test, OVR-FC, OVE-FC and OVE-sFC. Simulation data under the null hypothesis were generated from a Dirichlet distribution or a mixture of Dirichlet distributions, mimicking the general characteristics of non-negative molecular expression data (Chen *et al.*, 2021). Simulated MGs were introduced by assigning a complementary portion of genes as being uniquely expressed in only one subtype but not in any other, with the deviations from the ideal MG reference drawn randomly within certain degrees (Supplementary methods). We used both the partial receiver operating characteristic (pROC) curves and area under pROC curve (pAUC) to evaluate the accuracy of MG detection by COT and peer methods.

The experimental results are summarized in Figure 1B–E. Across various experimental settings (different number of subtypes, sample size per subtype and combination of Dirichlet distributions), COT consistently outperforms all peer methods in terms of higher detection power at an acceptable false-positive rate (FPR). Note that a stringent range of low FPR (0.01∼0.05) and a corresponding sufficient detection power (≥0.8) are emphasized here because the corresponding false discovery rate (FDR) would be problematic in real-world applications where large-scale multiple comparisons among many subtypes are encountered. Additional experimental results and discussions are presented in Supplementary information (Supplementary Figs. S1–S3, S8 and S10).

### 3.2 Verification of MG detected by COT on benchmark dataset

**Fig. 2.**
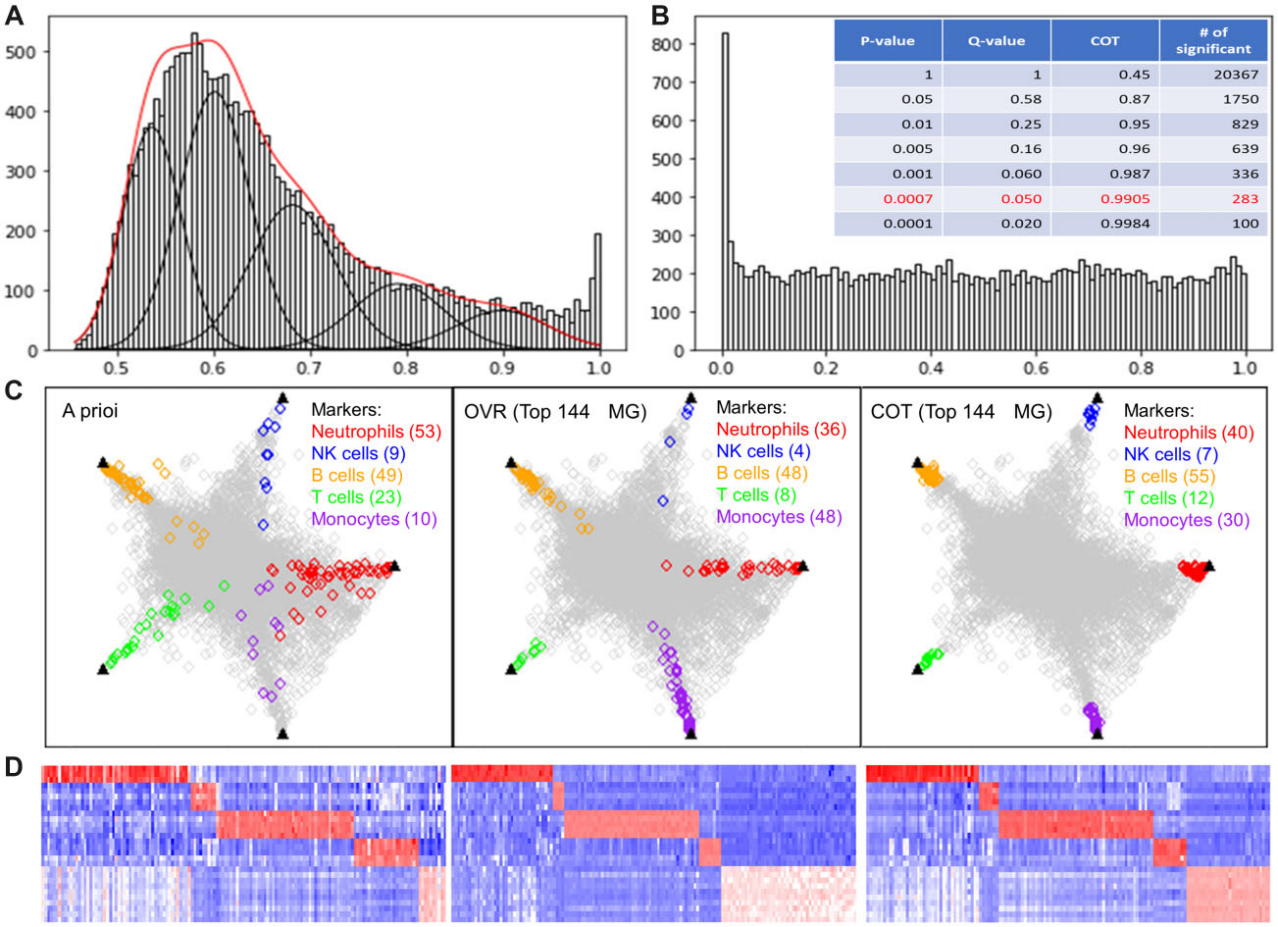
Verification of MG detected by COT on benchmark dataset (GSE28490). (**A**) The null distribution (histogram) superimposed with the FNM approximation using an FDR-embedded expectation-maximization algorithm (5 Gaussians, 4–6 iterations). (**B**) Empirical distribution of COT *P*-value and a concordance survey across different *P*-value threshold, *q*-value, COT threshold and number of accepted MG. (**C, D**) Simplex plots and heatmaps of MG (color-coded) detected by COT, OVR-test and *a priori* MG subset (column—protein, row—sample)

We verified the quality of MGs detected by COT on the benchmark real gene expression dataset (GSE28490 consisted of *K *=* *5 subtypes), in comparison with an *a priori* MG subset and also an MG subset detected by OVR *t*-test and Limma/EdgeR (the top performer reported in Section 3.1). The empirical distribution (histogram) of COT test statistic summarized over all genes is shown in Figure 2A, where the lower bound closely matches the expected value of $1/\sqrt{5}$. The converged FNM distribution that approximates the null distribution is superimposed and indicated by the red-colored curve. Figure 2B shows the empirical distribution of COT *P*-values over all genes, together with the corresponding *P*-value threshold, *q*-value, COT threshold and the number of accepted MG. The Venn diagram given in Supplementary Figure S9 shows the overlap between the top 144 MG detected by COT, OVR *t*-test and *a priori*.

The geometric proximity of the 144 color-coded MG detected by COT, OVR/Limma test and *a priori*, to the vertices (ideal MG references—black triangle) of scatter simplex is shown in Figure 2C and Supplementary Figure S11. The corresponding heatmaps are given in Figure 2D and Supplementary Figure S11. These results show that COT outperforms the best peer method and detects more ideal MG, for example, by their tighter grouping at the scatter simplex vertices. Quantitatively, the quality of the 144 MG detected by COT, measured by performance index $P_{1}$, is consistently higher than that of both peer methods and the *a priori* MG subset. Specifically, COT achieves a nearly perfect $P_{1,\;\mathrm{COT}}=0.019$, as compared with $P_{1,\;\mathrm{OVR}}=0.069$ by OVR test and $P_{1,\;\mathrm{known}}=0.118$ associated with the *a priori* MG subset reported in the literature. These improvements by COT correspond to a relative reduction of 72.5% over OVR test and 83.9% over *a priori* MG in terms of index $P_{1}$. Additional experimental results and discussions are presented in Supplementary information (Supplementary Figures S4 and S11—Limma).

We further performed benchmarking-based deconvolution experiments to demonstrate the increase in accuracy of deconvoluting cell types from the simulated mixtures using COT-MG versus the MG derived from OVR *t*-test and *a priori*. We emphasize that the complementary role of COT to existing methods refers to the ability of COT to both recruit more ideal and eliminate less-ideal MG. We compared the deconvolution accuracy using MG separately obtained by different methods instead of combined MG. The simulated bulk expression profiles were generated by randomly mixing the five cell types from the benchmark dataset in a total of 100 trials. Supplementary Table S2 summarizes the root-mean-square-error (RMSE) and sample-wise correlation between the COT-MG estimated and ground truth mixing proportions, showing that COT-MG achieves the lowest RMSE and highest sample-wise correlation, as compared with MG derived by OVR *t*-test and *a priori*.

### 3.3 Case study of detecting *de novo* MG on proteomics data of vascular specimens

**Fig. 3.**
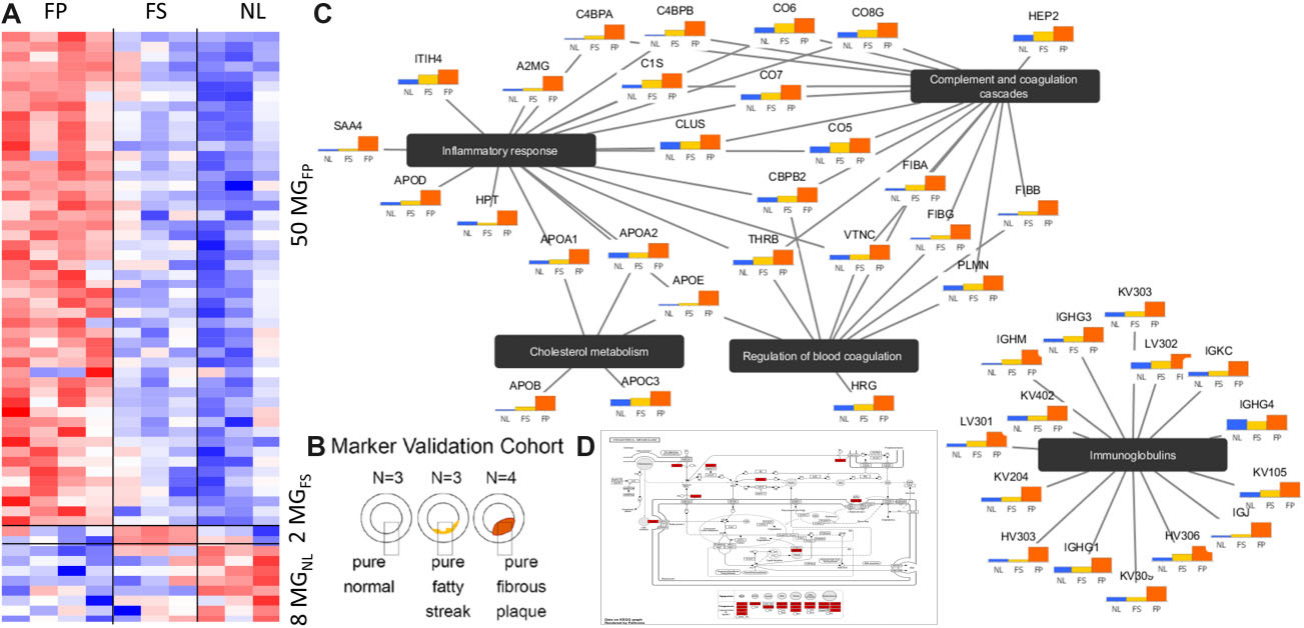
Case study of detecting *de novo* protein MG using proteomics data acquired from vascular specimens of three tissue subtypes. (**A, B**) Top 60 protein markers detected by COT on ‘pure’ vascular specimens (column—sample, row—protein). (**C**) The associated top enriched pathways in a perspective view. (**D**) KEGG map of cholesterol metabolism pathway enriched with COT FP-MG

To further demonstrate the utility of the COT method, we applied the COT tool to detect *de novo* tissue-specific MG proteins using two independently acquired mass spectrometry-based proteomic datasets (Fig. 3). The first experimentally acquired proteomics dataset was obtained from a cohort (*n* = 10) of ‘pure’ fibrous plaque (FP), fatty streak (FS) and normal (NL) vascular specimens (Fig. 3B; Parker *et al.*, 2020). Accordingly, COT detected 50 FP, 2 FS and 8 NL markers, respectively, with the heatmap shown in Figure 3A. These MG proteins are highly consistent with the MG detected by tissue deconvolution on a much larger cohort (Herrington *et al.*, 2018). The results of enrichment analysis are reported briefly in Figure 3C and D. The KEGG maps of FP markers show that nearly all components of the lipoprotein and immunoglobulin pathways have been detected (Fig. 3C). Intriguingly, two FS markers have previously been reported to be functionally involved in vascular pathology and atherosclerosis progression. Additional experimental results and discussions are presented in Supplementary iinformation.

Supplementary Table S1 provides a list of the top-ranked 60 MG proteins detected by COT using the proteomics data from pure samples. Analysis of the ‘pure’ specimen dataset detected proteins enriched in most of the same pathways that we reported previously (Parker *et al.*, 2020), including many of the immunoglobulins (indicative of immune activation and B-cell involvement), complement factors (indicative of immune activity and inflammation), protein degradation and many of the apolipoproteins that typically shuttle cholesterols back and forth between the liver and periphery; there is also evidence for local production by tissues including preliminary data demonstrating lipoprotein mRNA expression in LAD and AA tissues (unpublished data). The top KEGG map (Fig. 3D) shows that almost all components of the lipoprotein molecules have been detected. The Complement network diagram (Supplementary Fig. S5) shows that proteins involved in the later phase of complement activation seem to dominate the FP markers, which may have some functional significance.

The gene functions associated with the selected FS markers offer potential mechanistic insights. For example, GBB2 acts in concert with G-protein receptors including the angiotensin II type 1 receptor and beta-adrenergic receptor that are key regulators of some vascular pathologies. UGDH is an enzyme involved in the synthesis of glycosaminoglycans (GAGs) and an overall decrease in GAGs occurs as atherosclerosis progresses. In early stage, atherosclerotic lesions like FS a transient spike in some GAGs is seen that then regresses as the lesion progresses. Upregulation of UDGH, which may be rate limiting in GAG biosynthesis, also may drive selective and/or general GAG synthesis.

The normal marker NL proteins may also provide some useful biological insights based on their functions as annotated in the Uniprot database. For example, K2C8 is a cytoskeletal keratin involved in the contractile apparatus of striated muscle and may also play a similar role in linking the contractile apparatus of SMC to the desmosome (thus, involved in normal SMC function). MYL9 is also involved in SMC contraction and LIMS1 is important for cell–cell adhesion and cell survival via integrin signaling. Thus, SMCs appear to be linking to each other in their normal manner. Other MG are involved in general maintenance functions like endoplasmic reticulum folding and ribosomes, such as the normal markers of contractile apparatus, adhesion and extracellular matrix (K2C8, MYL9, LIMS1, SPRL1 and SPON1), and of protein synthesis, folding and quality control (RL11, HSP74 and DJC10). Supplementary Figure S6 shows the geometric proximity of the top MG to the vertices of the scatter simplex.

The second dataset is acquired from a cohort (*n* = 78) of heterogeneous vascular specimens containing mixed FP, FS and NL subtypes (Herrington *et al.*, 2018). The experimental results show that the detected two sets of MG are highly consistent across these two datasets. The objective of detecting MG using the second dataset acquired from ‘heterogeneous’ rather than ‘pure’ specimens is to cross-validate the MG detected from a small ‘pure’ sample cohort using an independent and much larger ‘heterogeneous’ sample cohort. We first applied a state-of-the-art unsupervised deconvolution tool, Convex Analysis of Mixtures (CAM; Chen *et al.*, 2020), to identify the ‘transformed’ reference of an ideal MG in the scatter space—the vertices of the scatter simplex. We then conducted COT in the scatter simplex of heterogeneous specimen dataset. Table 1 shows the consistency between the MG detected from the pure and heterogeneous samples. Note that FS is considered a ‘transitional’ subtype between NL and FP, and therefore the crosstalk among FS markers and NL and FP is expected (Herrington *et al.*, 2018; Parker *et al.*, 2020).

**Table 1.** Consistency between the top 60 MG detected by COT from purified specimens and by CAM from bulk tissues (51 FP-MG, 2 FS-MG, 7 NL-MG)

		COT based on pure specimen		
CAM based on bulk	Top 60 MG	MG_FP_ (51)	MG_FS_ (2)	MG_NL_ (7)
	Subtype 1	42	0	0
	Subtype 2	9	2	1
	Subtype 3	0	0	6

## 4 Discussion

The COT Python package provides an accurate and efficient software tool for data-driven MG detection among many subtypes. The proposed test statistic $\cos\left(s\left(i\right),\;{\hat{e}}_{k}\right)$ matches exactly the definition of MG and permits the formulation of a one-sample test. Conceptually, COT takes a more rigorous approach than looking at OVR fold change or performing *t*-tests (Chen *et al.*, 2021; Delaney *et al.*, 2019; Supplementary information). The experimental results show that COT consistently outperforms peer methods. While the case study here involves only transcripts and proteins, the COT method and software tool are readily applicable to other omics data types.

The null distribution plays a crucial role in large-scale multiple testing. However, because the number of pure subtype samples is often very small and non-MG patterns are often highly complex and intrinsically data-dependent, classical schemes to estimate the null distribution in a two-sample test setting are impractical (Chen *et al.*, 2021) or even inappropriate (Efron, 2004). A reasonable assumption is that the observed data can show the null distribution when a significant majority of features are associated with the null hypothesis (Efron, 2004). Thus, to ease the contamination of true MG in one-sample test, we proposed and implemented an FDR-guided iterative approximation of the null distribution (Supplementary information).

Furthermore, in large-scale multiple testing, a unified null distribution is widely adopted, where the test statistics are normalized by estimated sampling standard deviation. Because COT uses a cosine score function that is norm (magnitude) invariant yet different from the correlation function, variance stabilization is achieved automatically. Moreover, since the test statistic $\cos\left(s\left(i\right),\;{\hat{e}}_{k}\right)$ is calculated in reference to knowledge of the alternative hypothesis (*i.e.*, ${\hat{e}}_{k}$ represents the ideal MG), the resulting *P*-values are more meaningful (Efron, 2004).

Intended to identify MG using clustered single-cell data, an interesting yet novel method COMET has been specifically designed to exploit OVR gene-cluster enrichment analysis (Delaney *et al.*, 2019). However, some concerns remain with the use of pairwise OVR formulation that is limited intrinsically by its hypergeometric test (Chen *et al.*, 2021). For example, if a gene is highly enriched in both the cluster of interest and another ‘small’ cluster in the rest, this gene would be considered significant by OVR enrichment analysis but a poor marker gene (non-unique) for the cluster of interest. In contrast, this gene will not be picked up by COT because the cosine score compares the entire cross-subtype expression vector with the definition vector of MG.

When the number of ideal MG for different subtypes is significantly imbalanced, we suggest constructing subtype-specific null distributions to detect MG for each subtype separately. Accordingly, the test statistic for specific subtype *k* is given by

(5) $$\begin{array}{c} t_{\text{COT}}(i_{\text{MG},\;k})=\cos\left(s\left(i\right),\;{\hat{e}}_{k}\right)=\frac{s_{k}\left(i\right)}{\sqrt{\sum_{j=1}^{K}{\left[s_{j}(i)\right]}^{2}}},\# \end{array}$$

where detected MG for some subtype(s) may be suboptimal (less ideal) but still biologically informative. Moreover, the test statistic (5) can be used to detect subtype signature genes (SSG; Chen *et al.*, 2021), with the heatmap of SSG shown in Supplementary Figure S7 corresponding to the same case study reported in Figure 3A. This alternative scheme also reduces the contamination of true MG in the one-sample test because the true MG of other subtypes becomes non-MG for the specific subtype.

Alternatively, a molecularly distinct subtype may be characterized by molecular features that are uniquely silent in the cell or tissue subtype of interest but in no others—so-called subtype-downregulated genes (SDG). Mathematically, an SDG of subtype *k* is defined as a gene being silent only in subtype *k* but not in any other subtypes, approximately

(6) skiSDG, k≈0,sl≠kiSDG, k≫0,#

and a modified COT test statistic can be designed to detect SDG given by

(7) $$\begin{array}{c} t_{\text{COT}}(i_{\text{SDG}})=\underset{1\leq k\leq K}{\mathrm{argmax}}\cos\left(s\left(i\right),\;{\hat{e}}_{k}⊕\vec{1}\right)=\underset{1\leq k\leq K}{\mathrm{argmax}}\frac{\sum_{j\neq k}s_{j}(i)}{\sqrt{(K-1)\sum_{j=1}^{K}{\left[s_{j}(i)\right]}^{2}}},\# \end{array}$$

where ⊕ is the exclusive disjunction XOR operation, and $1/\sqrt{K-1}<t_{\text{COT}}(i)<1$.

## Funding

This work has been supported by the National Institutes of Health [grant numbers HL111362-05A1, HL133932, NS115658-01] and the Department of Defence under Grant W81XWH-18-1-0723 [BC171885P1].

*Conflict of Interest*: none declared.

## Data Availability

Benchmark dataset used in this paper can be downloaded from Gene Expression Omnibus (https://www.ncbi.nlm.nih.gov/geo/) under the accession number: GSE28490.

## Supplementary Material

vbac037_Supplementary_Data
