# Supplementary material for: COT: an efficient and accurate method for detecting marker genes among many subtypes
Source: Bioinform Adv. 2022 May 27;2(1):vbac037. doi: 10.1093/bioadv/vbac037 (PMC9163574; doi:10.1093/bioadv/vbac037)
Supplement: vbac037_Supplementary_Data [file vbac037_supplementary_data.docx]

Supplementary Information

**COT: an efficient and accurate method for detecting marker genes among many subtypes**

Yingzhou Lu, Chiung-Ting Wu, Sarah J. Parker, Zuolin Cheng, Georgia Saylor, Jennifer E. Van Eyk, Guoqiang Yu, Robert Clarke, David M. Herrington, Yue Wang

**Contents**

[Introduction 2](#_Toc99196093)

[Method 4](#_Toc99196094)

[Results 6](#_Toc99196095)

[Discussion 7](#_Toc99196096)

[Scripts 8](#_Toc99196097)

[Supplementary Tables 10](#_Toc99196098)

[Supplementary Figures 12](#_Toc99196099)

[References 18](#_Toc99196100)

## Introduction

Many data-driven methods have been developed to detect subtype signature genes (SSG), that is, subtype-specific differentially expressed genes (Chen, et al., 2021). These methods include One-Versus-Rest (OVR) test (Delaney, et al., 2019; Yu, et al., 2010), One-Versus-One (OVO) test (Hunt, et al., 2019), and most recently One-Versus-Everyone (OVE) test (Wang, et al., 2016). The concept of SSG is defined as being most-upregulated in only one subtype but not in any other, that is, we considered any case with at least 'two-equal winners’ as non-SSG. To address the critical problem of the absence of accurate methods to detect SSG, we have previously proposed, tested and applied One-Versus-Everyone Fold Change (OVE-FC) test (Yu, et al., 2010; Yu, et al., 2011) and more recently blended OVE/OVO permutation/t-test (Chen, et al., 2021). We have demonstrated real biomedical utilities of these methods on gene expression and proteomics data for the purpose of characterizing or classifying complex subtypes. These applications have led to novel findings and hypotheses (Herrington, et al., 2018; Parker, et al., 2020).

Clearly, the requirements for being a marker gene (MG) are much more stringent than for an SSG. We and others have recognized that the test statistics used by most existing methods do not exactly satisfy MG definition (Chen, et al., 2021; Hunt, et al., 2019; Yu, et al., 2010). While the COT method reported here evolves from our recent work on detecting SSG, the COT framework is fundamentally different from most existing methods. First, COT addresses MG detection among many subtypes, but not SSG. Second, COT proposes a novel test statistic - the cosine similarity between a gene's cross-subtype expression pattern and the definition of an ideal MG. Third, COT formulates MG detection as a one-sample test rather than two-sample test (Efron, 2004). We emphasize that the expression patterns under the null hypothesis (non-MG) is highly 'complex', and no existing permutation scheme can effectively estimate the null distribution of a given test statistic. Conversely, summarization of the information among numerous null features, after proper 'scaling', provides the possibility of estimating the null distribution of MG test statistic without often untraceable sample permutation.

We would like to use a toy example to illustrate the expression patterns of ideal MG according to the given definition (red points the illustration below) and all possible expression patterns of DEG in various forms (yellow and blue except green points), illustrating the complexity of the null hypothesis with respect to MG.


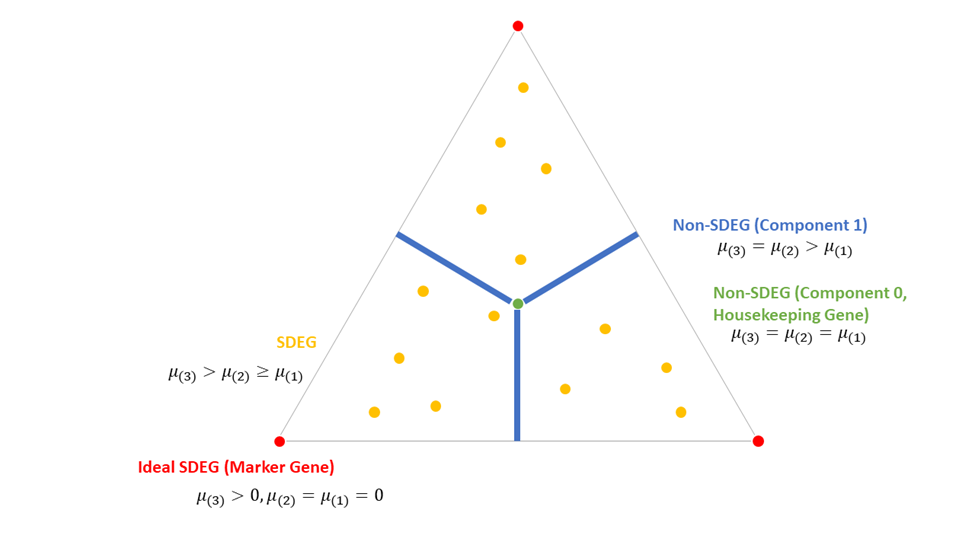


Consider four subtypes, ‘1’ denotes high expression, and ‘0’ denotes low expression. Then among all possible expression patterns in four subtypes, ideal MG are (0,0,0,1) (0,0,1,0) (0,1,0,0) (1,0,0,0). and non-MG are (0,0,0,0) (1,1,1,1) (0,0,1,1) (0,1,0,1) (0,1,1,0) (1,0,0,1) (1,0,1,0) (1,1,0,0) (0,1,1,1) (1,0,1,1) (1,1,0,1) (1,1,1,0).

Since ANOVA uses the null hypothesis focusing solely on (0,0,0,0) (1,1,1,1), i.e., the samples in all four subtypes are drawn from the same distribution, it may wrongly detect (0,0,1,1) (0,1,0,1) (0,1,1,0) (1,0,0,1) (1,0,1,0) (1,1,0,0) as MG. In fact, these are false positive MG because they are actually the subtype-nonspecific classic DEGs (differentially expressed across any of the subtypes). Similar situation would occur for pairwise OVR t-test particularly when the ‘2^nd^ 1’ in the rest is brought down by the majority of ‘0s’.

## Method

***Alternative definition of MG***

The proposed definition of MG is adopted from the literature (Chikina, et al., 2015; Delaney, et al., 2019; Hunt, et al., 2019; Kuhn, et al., 2012; Wang, et al., 2021; Zhong, et al., 2013). We acknowledge that there are some alternative definitions and our adopted definition is not a universal standard in the field (Dimitrakopoulou, et al., 2018; Sathe, et al., 2020).

We would like to emphasize that one advantage of the proposed MG definition is that they are individually and uniquely/exclusively enriched in a particular subtype, thus regardless of their expression level(s) but only identities, they can be readily used in facilitating deconvolution or classification (Kuhn, et al., 2011; Wang, et al., 2016; Yates and Boeva, 2022). This has been mathematically proven via Theorem 1 in (Wang, et al., 2016). In contrast, for the use of some alternatively defined MGs (e.g., a set of signature or discriminatory genes) in enabling deconvolution or classification, their specific and reliable expression levels are often necessarily required in addition to their identities (Dimitrakopoulou, et al., 2018; Newman, et al., 2015).

***Data preprocessing***

Prior to COT analysis, preprocessing procedures should be applied to remove or reduce technical or experimental variations in datasets, including within/between-sample normalization, batch effect correction, or confounder adjustment *etc*. when applicable. Many existing normalization pipelines or packages may be used, including RMA, PLIER, SWAN, BMIQ and more recently RPKM, DESeq2, TMM, and TCC. Batch effect is the systematic error introduced by the time- and site-dependent experimental variations. ComBat (Li, et al., 2006) is a popular and effective method to remove batch effects.

***Refinement of the null distribution***

To ease the contamination of true MG in a one-sample test (right-peak in Figure 2A), we proposed and implemented an FDR-guided iterative approximation of the null distribution. We use a mixture of *K* normal distributions (finite normal mixture – FNM model) to approximate the empirical $t_{\text{COT}}(i)$ distribution (Efron, 2004; Equihua, 1988). The FNM distribution is initialized by agglomerative clustering and then estimated by the expectation-maximization (EM) algorithm (Wang, et al., 1998). We then improve the approximation by an FDR-guided iterative estimation strategy, where the same number of ‘falsely accepted’ MG, as predicted by the q-value estimate, are randomly/uniformly removed from the next iteration of the approximation (Efron, 2004; Storey and Tibshirani, 2003). In our experiments, this procedure converges to a stationary point usually within 5~20 iterations with adjusted sequential cut-off p-values of 0.001, 0.005, 0.01, and 0.05.

***Alternative performance index***

The quantitative index $P_{1}$ for measuring the quality of MG is rooted in the earlier $E_{1}$ index in independent component analysis (Hyvärinen, et al., 2001), defined as

$$\begin{aligned} E_{1}=\sum_{j=1}^{K} \left( \sum_{i=1}^{K} \frac{\bar{s}_{j}\left( i_{\text{MG}} \right)}{\text{max}_{k}\bar{s}_{j}\left( k_{\text{MG}} \right)}-1 \right)+\sum_{i=1}^{M} \left( \sum_{j=1}^{K} \frac{\bar{s}_{j}\left( i_{\text{MG}} \right)}{\text{max}_{k}\bar{s}_{k}\left( i_{\text{MG}} \right)}-1 \right), \end{aligned}$$

where $\bar{s}_{j}\left( i_{\text{MG}} \right)$ is the averaged value of MG within subtype $j$. Again, the $E_{1}$ index attains its minimum value zero for an ideal set of averaged MG.

***Additional description of simulation data***

For Figure 1B, the design settings are: data distribution under the null hypothesis (non-MG)

follows a Dirichlet distribution [1 1 1], data distribution under the alternative hypothesis (MG) follows a truncated/non-negative Gaussian distribution (mean: 0, variance: standard deviation 0.20), number of total genes = 5000, number of subtypes = 3, number of samples per subtype = 3, number of non-MGs = 4500, and number of MGs = 500 (10%).

For Figure 1C, the design settings are: data distribution under the null hypothesis (non-MG)

follows a Dirichlet distribution [1 1 1], data distribution under the alternative hypothesis (MG) follows a truncated/non-negative Gaussian distribution (mean: 0, variance: standard deviation 0.20), number of total genes = 5000, number of subtypes = 5, number of samples per subtype = 3, number of non-MGs = 4500, and number of MGs = 500 (10%).

For Figure 1D, the design settings are: data distribution under the null hypothesis (non-MG)

follows a Dirichlet distribution [1 1 1], data distribution under the alternative hypothesis (MG) follows a truncated/non-negative Gaussian distribution (mean: 0, variance: standard deviation 0.20), number of total genes = 5000, number of subtypes = 3, number of samples per subtype = 4, number of non-MGs = 4500, and number of MGs = 500 (10%).

For Figure 1E, the design settings are: data distribution under the null hypothesis (non-MG)

follows a mixture of a Dirichlet distribution [2 2 2] and a rotated Dirichlet distribution [1.5 1.5 1.5]*[0, 0.5, 0.5; 0.5, 0, 0.5; 0.5, 0.5, 0], data distribution under the alternative hypothesis (MG) follows a truncated/non-negative Gaussian distribution (mean: 0, variance: standard deviation 0.20), number of total genes = 5000, number of subtypes = 3, number of samples per subtype = 3, number of non-MGs = 4500 (3600, 900), and number of MGs = 500 (10%).

## Results

Type 1 error rate assessment is crucial for methods that detect MG based on their p-values, since if the type 1 error rate is either too conservative or too liberal, the p-value loses its intended meaning and does not reflect the actual false positive rate. Our experimental results on assessing type 1 error rate are shown in Figure S1, where the distribution of p values under the null hypothesis is approximately uniform indicating a good match between the empirical false positive rate and the theoretical type 1 error rate.

For the same dataset and result shown in Figure 2, we performed additional comparisons with other peer methods, assessed by the $E_{1}$ index. As expected, the quality of MG identified by COT is significantly higher than that of additional peer methods, achieving the lowest $E_{1,\mathrm{COT}}=0.779$ as compared to $E_{1,OVE/OVO}=2.760$ by OVE/OVO t-test and $E_{1,\mathrm{known}}=4.232$ associated with known markers reported in literature. These improvements correspond to a 76.8% error reduction as compared to $P_{1,OVE/OVO}=0.082$ by OVE/OVO t-test and an 83.9% error reduction as compared to $P_{1,\mathrm{known}}=0.118$ associated with known markers reported in the literature.

## Discussion

Fundamental to the success of the COT method is the newly-proposed test statistic $\cos\left( \boldsymbol{s}\left( i \right), {\hat{\boldsymbol{e}}}_{k} \right)$ that measures directly the distance between the cross-subtype expression patterns of gene *i* and the ideal MG of subtype *k* in scatter space. The known cross-subtype expression patterns of ideal MG associated with the alternative hypothesis permit the use of a one-sample test to detect significant MG, making the resulting p-values more meaningful in the framework of Bayes hypothesis testing and avoiding the caveat in general significant tests with no knowledge of the alternative hypothesis (Efron, 2004).

More importantly, the COT framework is efficient in that neither OVE set intersection nor intractable sample permutation is needed in estimating the null distribution (Chen, et al., 2021; Hunt, et al., 2019), where a significant majority of genes are assumed to be associated with the null hypothesis. The theoretical null may fail in some cases, which is not completely wrong but needs adjustment. In our study, we adopted a mixture of five normal distributions FNM to approximate the empirical COT distribution (Equihua, 1988; Wang, et al., 1997). Moreover, instead of modeling only the null distribution and using FDR-guided iterative estimation strategy, one can also simultaneously model and estimate both the null and alternative distributions using a proper mixture distribution when the number of features associated with the alternative hypothesis is sufficient (Wang, et al., 1998).

Indeed, COMET is an interesting yet novel method that is intended to identify marker genes and specifically designed to exploit clustered single-cell data and OVR gene-cluster enrichment analysis. Specifically, COMET uses the XL-mHG test to adaptively binarize gene expression data and performs a gene-specific maximum gene-cluster enrichment analysis, assessing the extent to which a gene could be a good marker for one particular cluster against the rest (OVR) (Delaney, et al., 2019).

The magnitude-invariant cosine score in COT aims similarly as the XL-mHG test with adaptive binarization to be not as dependent on effect size as a t-test might be, and thus COT or COMET would theoretically perform better in this regard. However, some concern remains on the OVR formulation intrinsically limited by the hypergeometric test (Chen, et al., 2021). For example, if a gene is highly enriched in both the cluster of interest and another ‘small’ cluster in the rest, this gene would be considered significant by OVR enrichment analysis but a poor marker gene (non-unique) for the cluster of interest. In contrast, this gene will not be picked up by COT because the cosine score compares the entire cross-subtype expression vector with the definition vector of MG.

## Scripts

The scripts below show how to obtain the same results as in “Case studies” section with default parameter settings. More suggestions on parameter setting can be found in the package vignette.

**GSE28490**

#%%

from COT.COT import COT

#%%

## Download GSE28490

import GEOparse

gse = GEOparse.get_GEO(geo="GSE28490", destdir="./", silent=True)

#%%

## Select needed cell types

cellTypes = {"Neutrophils": [], "NK": [], "CD19+ B": [], "CD8+ T": [], "CD14+ monocytes": []}

sampleType = [] # cell type labels

for cell in cellTypes:

    for gsm in gse.phenotype_data.index.values:

        if cell in gse.phenotype_data["source_name_ch1"][gsm]:

            cellTypes[cell].append(gsm)

            sampleType.append(cell)

samples = [] # sample ID

for cell in cellTypes:

    samples = samples + cellTypes[cell]

data = gse.pivot_samples('VALUE')[samples] # selected raw data

#%%

## Compute COT cosine value

cot = COT(df_raw=data, logarithmic_data=True)

cot.generate_subtype_means(sampleType)

cot.generate_cos_values()

#%%

## Estimate p-value

cot.estimate_p_values()

#%%

## Obtain and plot the subtype markers on the simplex (the same one on the manuscript)

cot.obtain_subtype_markers(top=144)

cot.plot_simplex()

#%%

## plot heatmap of subtype markers

cot.plot_heatmap()

#%%

## or, use the COT pipeline

cot_pl = COT(df_raw=data, logarithmic_data=True)

cot_pl.cot_pipeline(subtype_label=sampleType, top=144)

#%%

## Compare OVO T-Test

import numpy as np

import pandas as pd

# Take log of the raw data

df_log = np.log2(cot.df_raw)

# Prepare to compute the t-statistics for every pair of subtypes

mean = df_log.apply(

        lambda x: np.array([np.mean(x[col])

            for col in cot.subtypes.values()]), axis=1)

var = df_log.apply(lambda x: np.array([np.var(x[col], ddof=1) for col in cot.subtypes.values()]), axis=1)

n = df_log.apply(lambda x: np.array([len(col) for col in cot.subtypes.values()]), axis=1)

# Find and compute the subtype with the highest t-statistics

idx = mean.apply(lambda x: np.argmax(x))

# The empty column 'score' and 'score_subtype' are prepared for other methods comparing with COT

cot.df_cos['score'] = pd.DataFrame({'mean': mean, 'n': n, 'var': var, 'idx': idx}) \

        .apply(lambda x: (x['mean'][x['idx']] - x['mean']) /

            np.sqrt(x['var'][x['idx']] / x['n'][x['idx']] + x['var'] / x['n']), axis=1) \

                .apply(lambda x: np.sort(x)).apply(lambda x: x[1])

cot.df_cos['score_subtype'] = idx.apply(lambda x: list(cot.subtypes.keys())[x])

#%%

## Obtain and plot the subtype markers from OVO t-test on the simplex (the same one on the manuscript)

# The 'scoreThre' is the threshold for 'score' column (higher better)

# When 'scoreThre' is not None, cosThre, pThre, and qThre are invalid automatically

cot.obtain_subtype_markers(top=144, scoreThre=0)

cot.plot_simplex()

#%%

## plot heatmap of subtype markers from OVO t-test

cot.plot_heatmap()

## Supplementary Tables

**Table S1.** List of top 60 MG detected by COT on proteomics data of pure samples.

| **Uniport** | **Gene** | **MaxCos** | **Subtype** |
| --- | --- | --- | --- |
| P01023 | A2MG | 0.984953 | FP |
| P35858 | ALS | 0.949705 | FP |
| P04114 | APOB | 0.932073 | FP |
| P05090 | APOD | 0.916104 | FP |
| P02649 | APOE | 0.940183 | FP |
| P04003 | C4BPA | 0.941963 | FP |
| P20851 | C4BPB | 0.889659 | FP |
| Q96IY4 | CBPB2 | 0.874947 | FP |
| P02671 | FIBA | 0.938695 | FP |
| P02675 | FIBB | 0.962863 | FP |
| P02679 | FIBG | 0.966014 | FP |
| P00738 | HPT | 0.973683 | FP |
| P01871 | IGHM | 0.932454 | FP |
| P01591 | IGJ | 0.987092 | FP |
| P19823 | ITIH2 | 0.926847 | FP |
| Q96PD5 | PGRP2 | 0.886458 | FP |
| P00747 | PLMN | 0.863939 | FP |
| P55058 | PLTP | 0.977319 | FP |
| O00391 | QSOX1 | 0.915904 | FP |
| P35542 | SAA4 | 0.967419 | FP |
| P04004 | VTNC | 0.926702 | FP |
| O60701 | UGDH | 0.843959 | FS |
| P00450 | CERU | 0.855635 | FP |
| P00734 | THRB | 0.862961 | FP |
| P01031 | CO5 | 0.841316 | FP |
| P01597 | KV105 | 0.879806 | FP |
| P01617 | KV204 | 0.911765 | FP |
| P01621 | KV303 | 0.891841 | FP |
| P01625 | KV402 | 0.912694 | FP |
| P01714 | LV301 | 0.916399 | FP |
| P01764 | HV303 | 0.965693 | FP |
| P01767 | HV306 | 0.900022 | FP |
| P01834 | IGKC | 0.887244 | FP |
| P01857 | IGHG1 | 0.859667 | FP |
| P01860 | IGHG3 | 0.888342 | FP |
| P01861 | IGHG4 | 0.905672 | FP |
| P01876 | IGHA1 | 0.938344 | FP |
| P02647 | APOA1 | 0.934599 | FP |
| P02652 | APOA2 | 0.90552 | FP |
| P02656 | APOC3 | 0.916256 | FP |
| P04196 | HRG | 0.854048 | FP |
| P04433 | KV309 | 0.956962 | FP |
| P05546 | HEP2 | 0.840869 | FP |
| P05787 | K2C8 | 0.888285 | NL |
| P07360 | CO8G | 0.874533 | FP |
| P08185 | CBG | 0.840799 | FP |
| P09871 | C1S | 0.849535 | FP |
| P10643 | CO7 | 0.890565 | FP |
| P10909 | CLUS | 0.84181 | FP |
| P13671 | CO6 | 0.861453 | FP |
| P19827 | ITIH1 | 0.861765 | FP |
| P24844 | MYL9 | 0.848724 | NL |
| P34932 | HSP74 | 0.843921 | NL |
| P48059 | LIMS1 | 0.8543 | NL |
| P62879 | GBB2 | 0.842642 | FS |
| P62913 | RL11 | 0.846606 | NL |
| P80748 | LV302 | 0.879469 | FP |
| Q14515 | SPRL1 | 0.848788 | NL |
| Q14624 | ITIH4 | 0.863949 | FP |
| Q9HCB6 | SPON1 | 0.90266 | NL |

**Table S2.** The RMSE and sample-wise correlation between the COT-MG estimated and ground truth mixing proportions in benchmark-based deconvolution experiments.

**
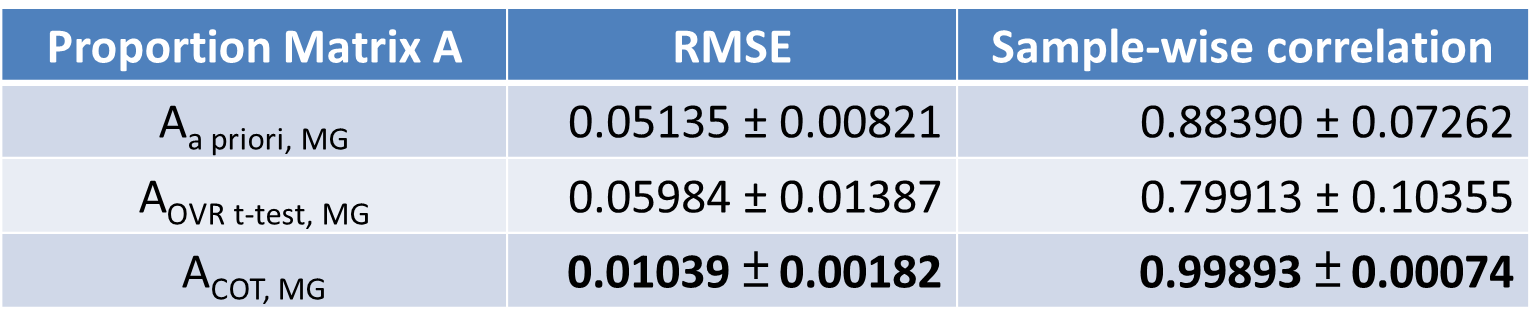
**

## Supplementary Figures


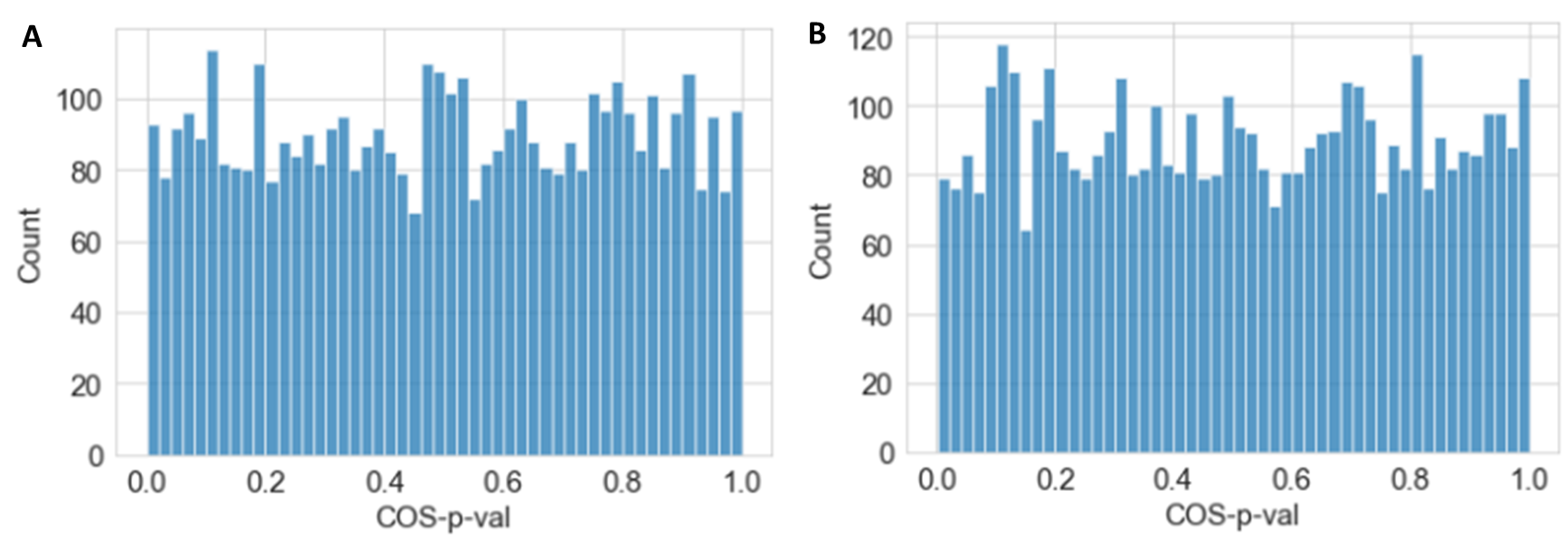


**Figure S1.** The histogram (empirical distribution) of COT p-values under the null hypothesis. (A) K=3, n=3x3, 4,500 genes. (B) K=5, n=3x5, 4,500 genes.


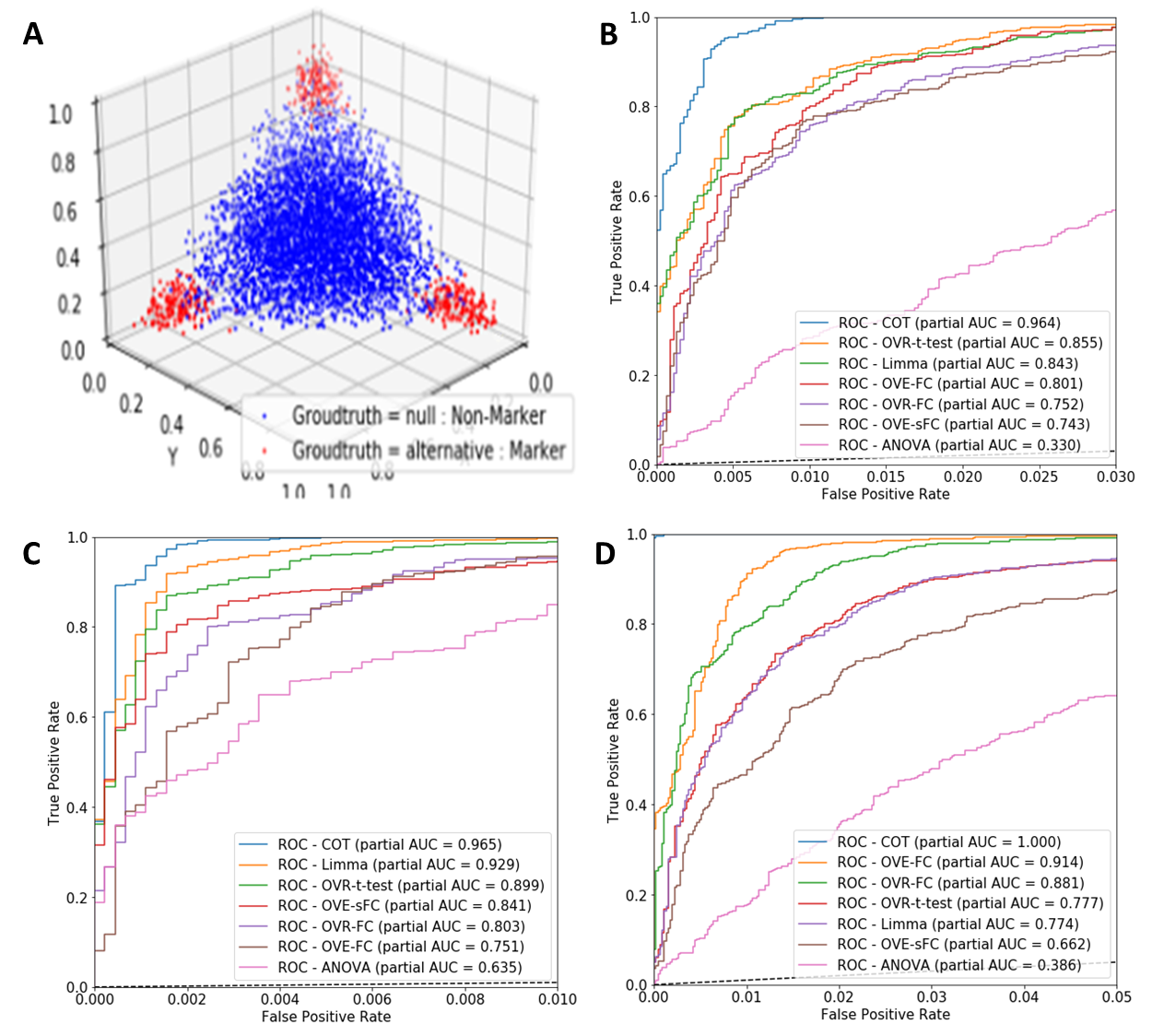


**Figure S2.** Evaluation and comparison of COT and peer methods (including Limma) using simulation data. (A) Simplex plot of a typical data matrix. Total number of genes 5,000. The Dirichlet distribution under the null hypothesis uses α=1, 2, and 3. (B-D) Additional pROC curves and pAUC values associated with COT and peer methods in the more-subtype (*K*=4, n=3×**4**), more-sample (*K*=3, n=**5**×3), and complex-null (mixture of rotated Dirichlet distributions) experimental settings, respectively.


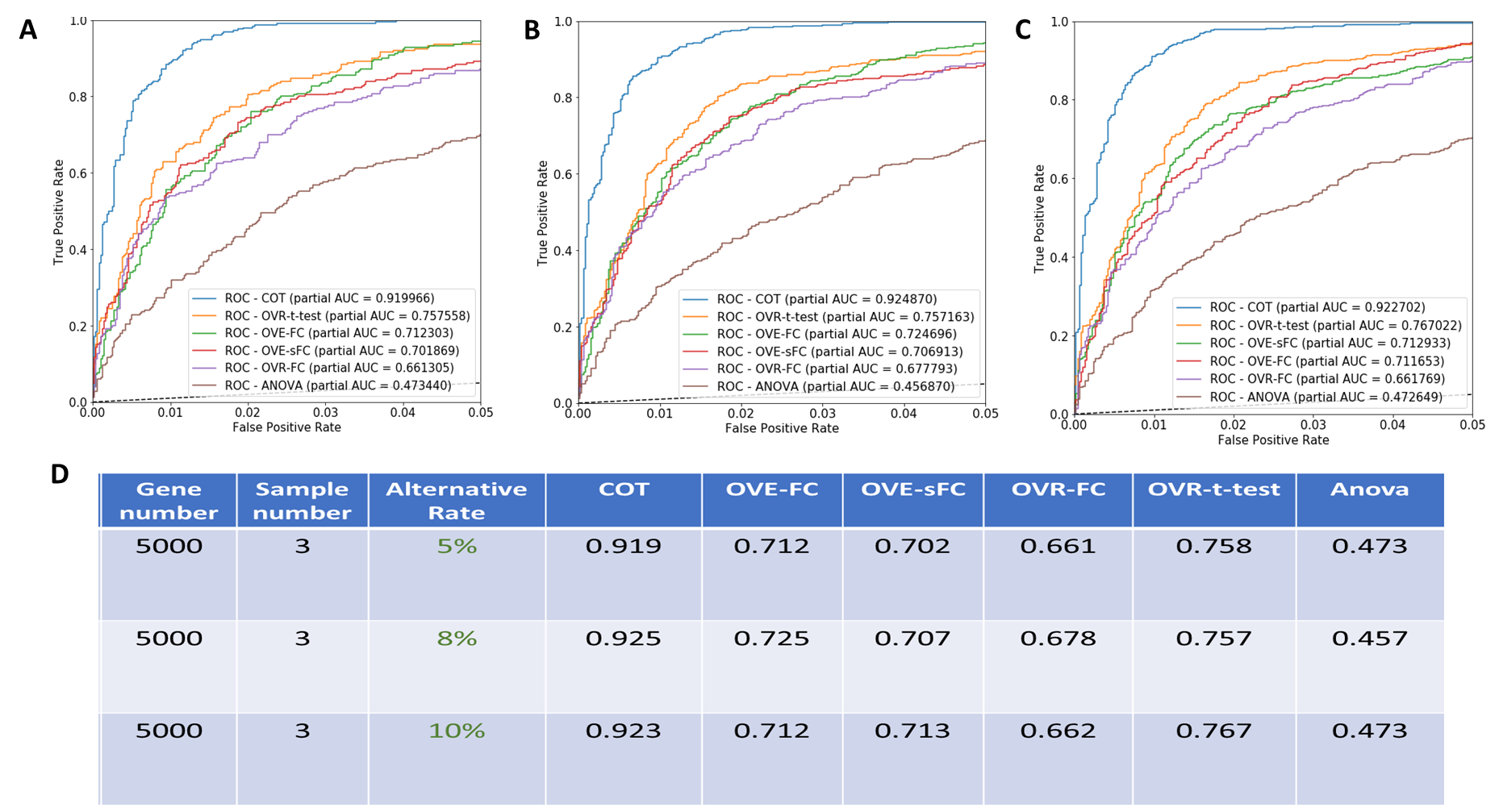


**Figure S3.** Evaluation and comparison of COT and peer methods using simulation data. Additional pROC curves and pAUC values associated with COT and peer methods in the experimental settings with varying mixture of MG and non-MG percentages, respectively. (A) 4,750 non-MG and 250 (5%) MG. (B) 4,600 non-MG and 400 (8%) MG. (C) 4,500 non-MG and 500 (10%) MG. (D) Summary table.


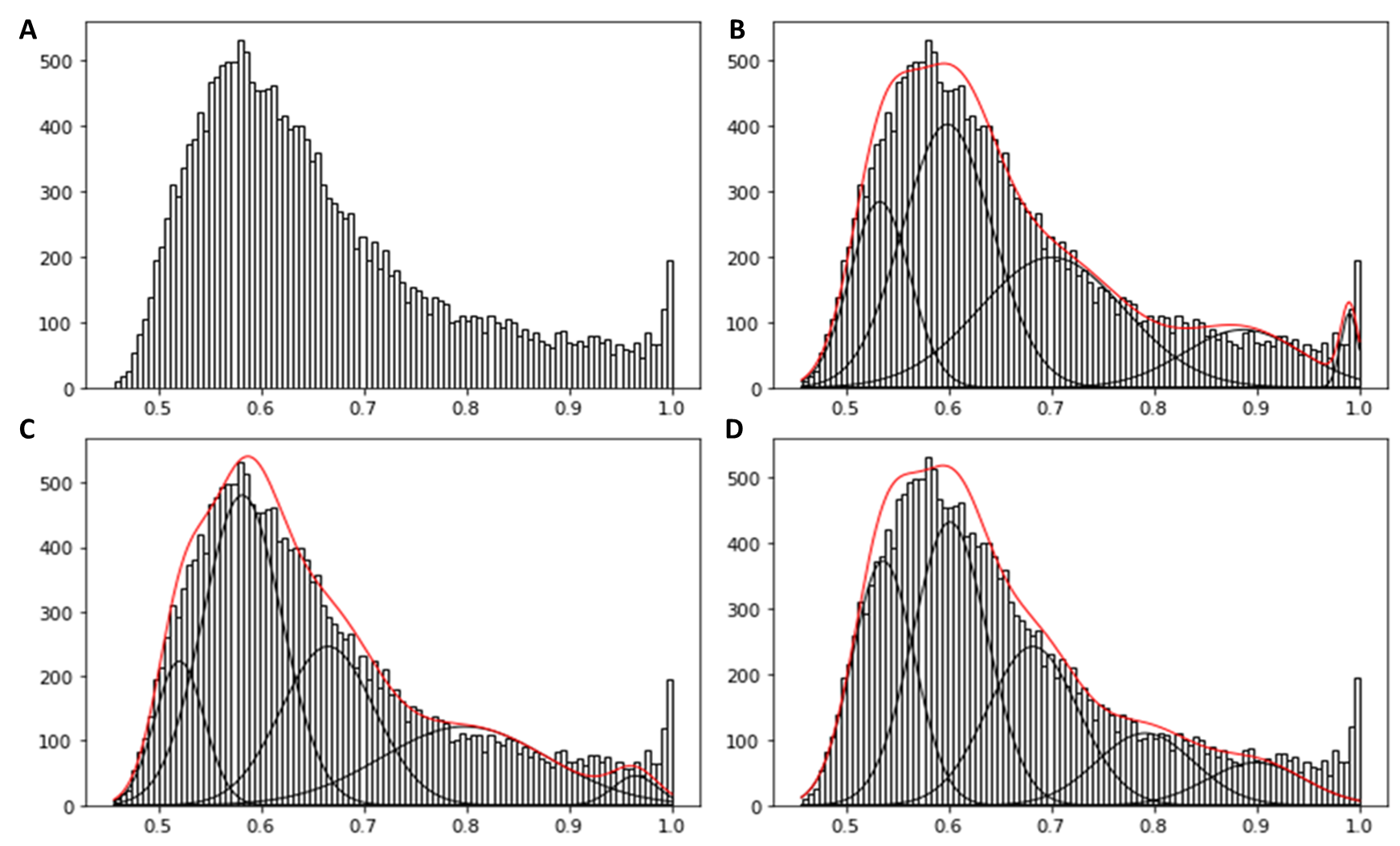


**Figure S4.** (A) The distribution (histogram) of COT test statistic in the GSE28490 dataset. (B) The initial fitting of COT distribution using a mixture of five normal distributions without FDR-guided adjustment, where the black curves are the estimated individual Gaussian kernels and the red curve is the overall mixture distribution. (C-D) The converged FNM approximation of the COT distribution using an FDR-guided iterative estimation strategy (left: 4 iterations, right: 6 iterations).


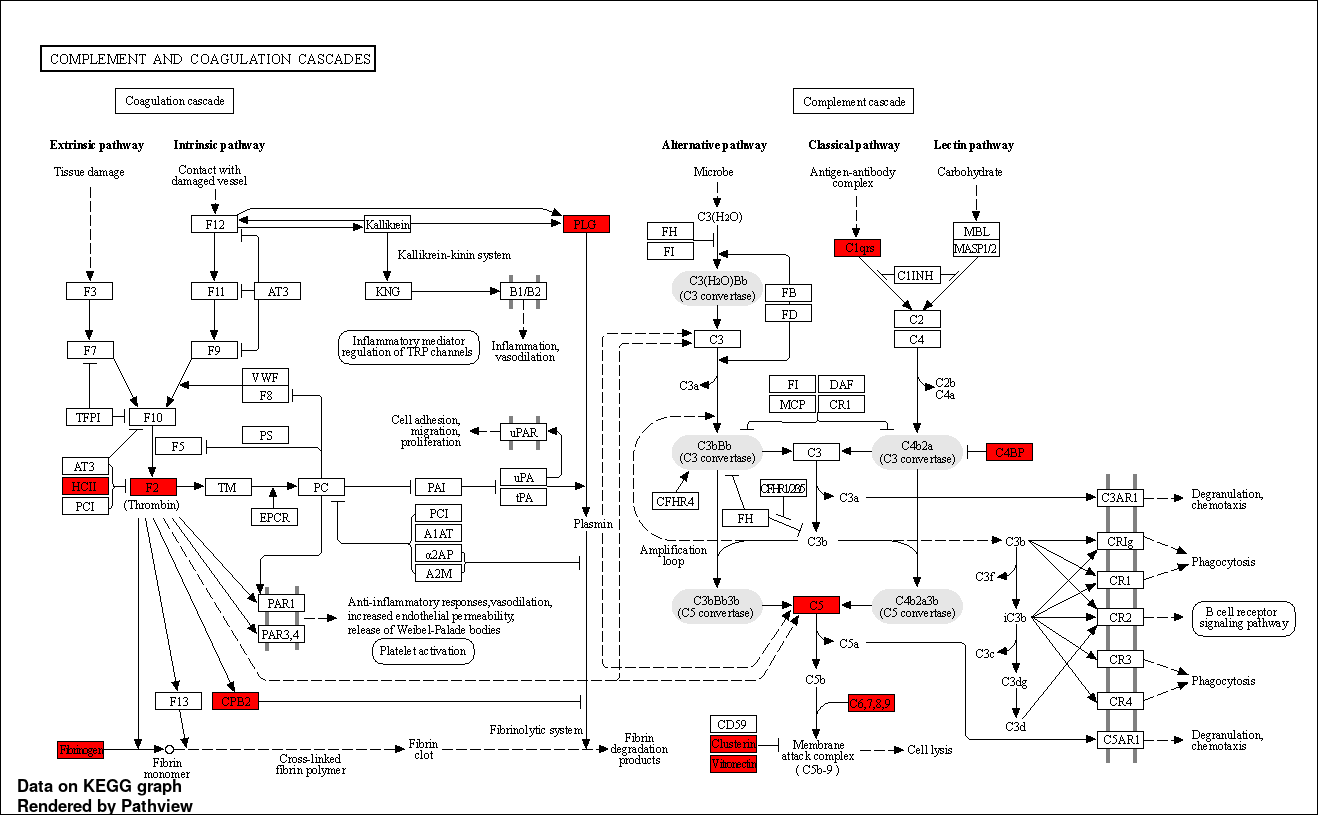


**Figure S5.** KEGG map of Complement and Coagulation pathway enriched with COT FP MG.


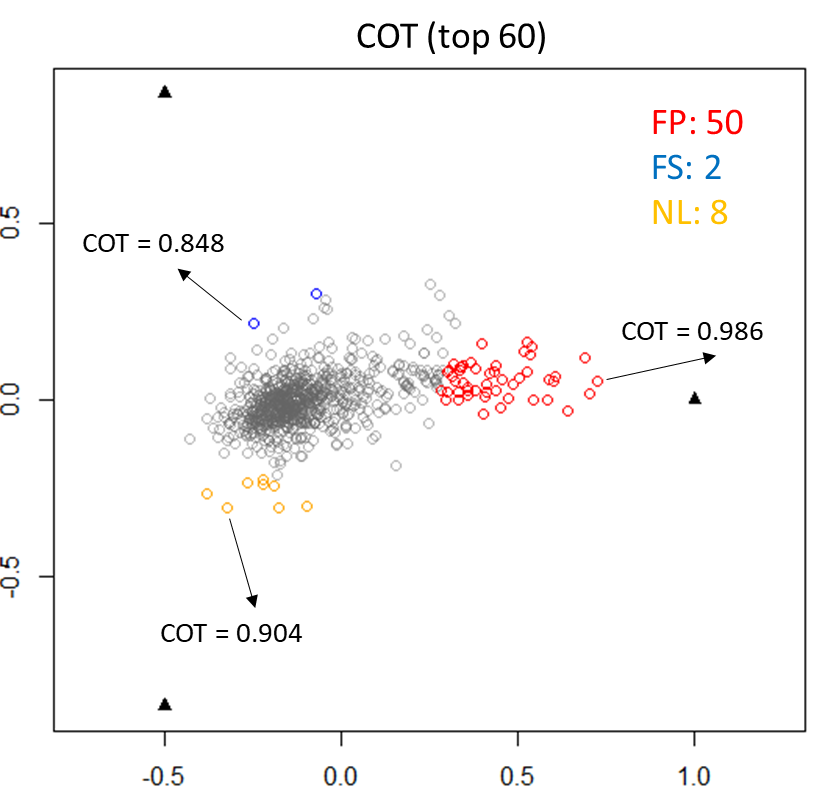


**Figure S6.** Scatter simplex of color-coded top 60 protein MG detected by CAM directly from heterogeneous samples.


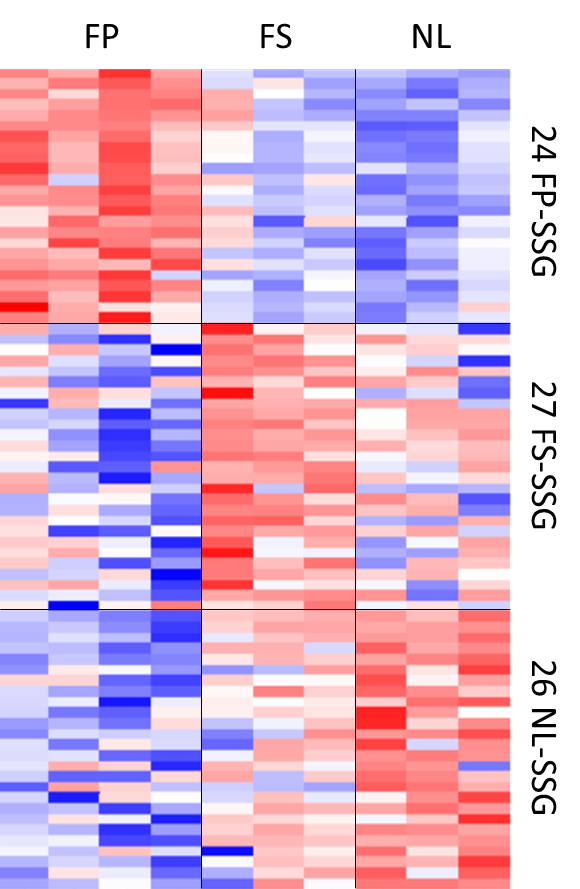


**Figure S7.** Heatmap of the color-coded top 77 subtype signature proteins (SSG) detected by subtype-specific COT (sCOT) on ‘pure’ vascular specimens (column – sample, row – protein).


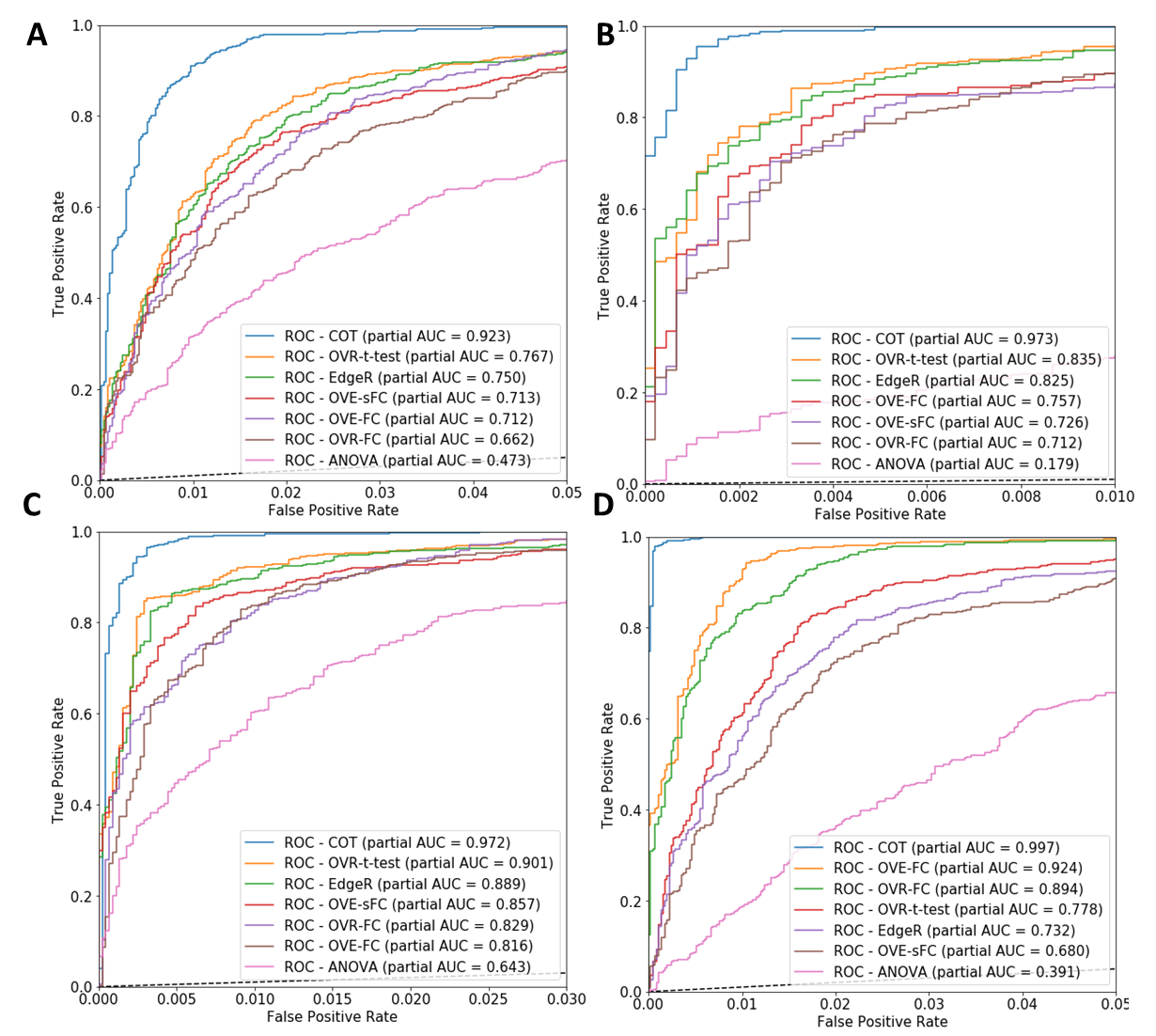


**Figure S8.** pROC curves and pAUC values associated with COT and EdgeR OVR test (supplementary to Figure 1BCDE) in the standard (*K*=3, *n*=3×3), more-subtype (*K*=5, *n*=3×5), more-sample (*K*=3, *n*=4×3), and complex-null (mixture of rotated and non-uniform Dirichlet distributions) experimental settings, respectively.


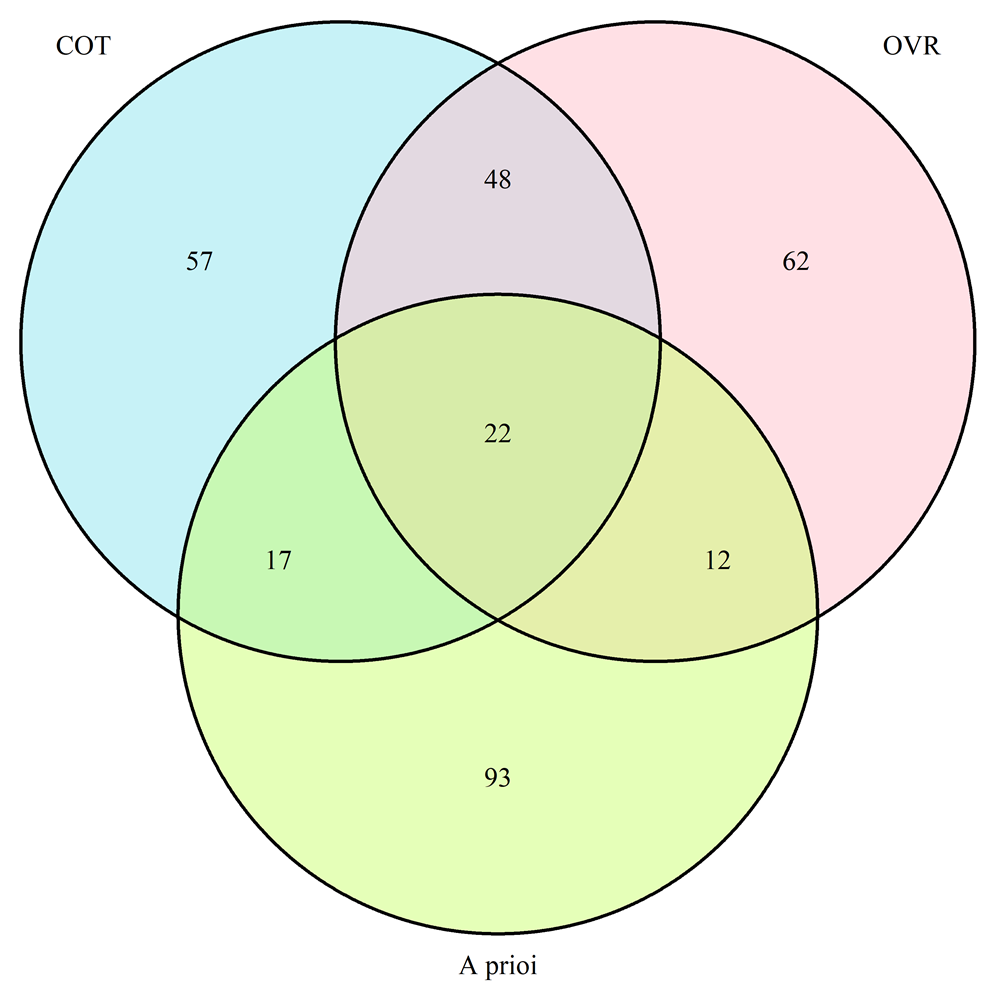


**Figure S9.** The Venn diagram showing the overlap between the top 144 MG detected by the COT, OVR t-test, and *a priori* in the benchmarking study.


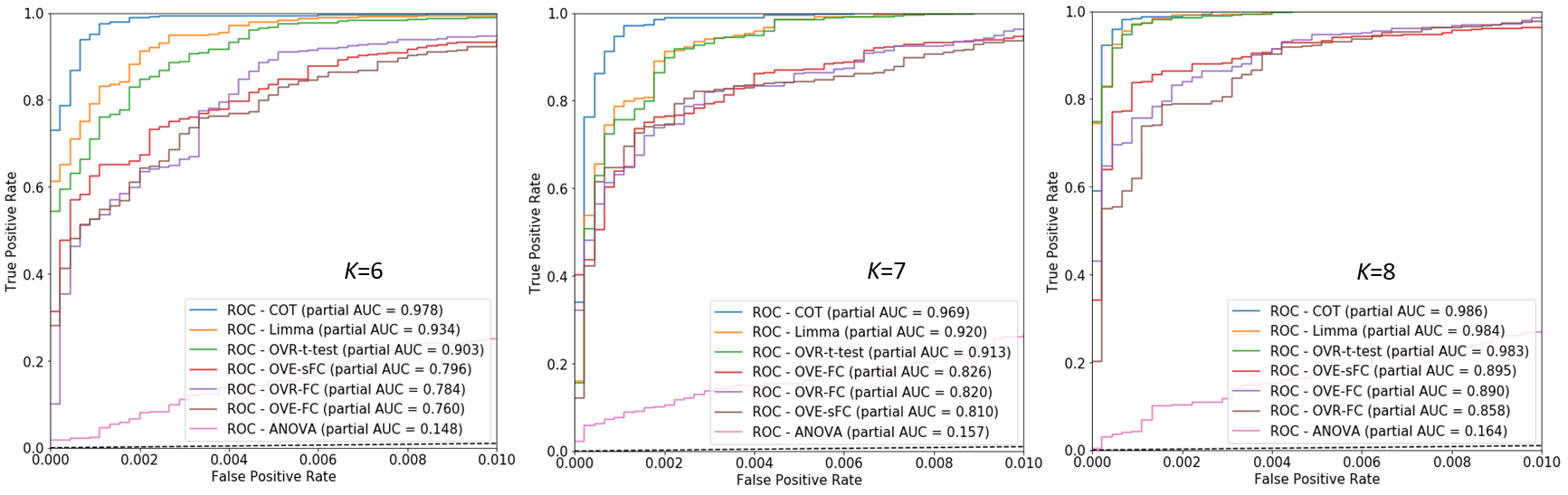


**Figure S10.** Evaluation and comparison of COT and peer methods using simulation data (supplementary Figure 1E). The pROC curves and pAUC values associated with COT and peer methods, under the complex-null (mixture of rotated Dirichlet distributions) experimental settings involving higher number of subtypes (*K* = 6, 7, 8, respectively).


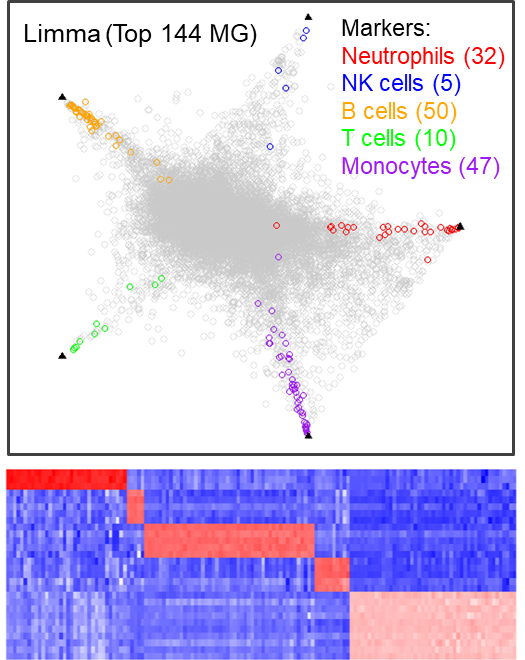


**Figure S11.** Verification of MG detected by COT on benchmark dataset (GSE28490). Simplex plots and heatmaps of top 144 MG (color-coded) detected by Limma OVR (column – protein, row – sample).

## References

Chen, L.*, et al.* Data-driven detection of subtype-specific differentially expressed genes. *Scientific Reports* 2021;11:332.

Chikina, M., Zaslavsky, E. and Sealfon, S.C. CellCODE: a robust latent variable approach to differential expression analysis for heterogeneous cell populations. *Bioinformatics* 2015;31(10):1584-1591.

Delaney, C.*, et al.* Combinatorial prediction of marker panels from single-cell transcriptomic data. *Mol Syst Biol* 2019;15(10):e9005.

Dimitrakopoulou, K.*, et al.* Deblender: a semi-/unsupervised multi-operational computational method for complete deconvolution of expression data from heterogeneous samples. *BMC Bioinformatics* 2018;19(1):408.

Efron, B. Large-scale simultaneous hypothesis testing: the choice of a null hypothesis. *Journal of the American Statistical Association* 2004;99(465):96-104.

Equihua, M. Analysis of finite mixture of distributions: a statistical tool for biological classification problems. *Comput Appl Biosci* 1988;4(4):435-440.

Herrington, D.M.*, et al.* Proteomic Architecture of Human Coronary and Aortic Atherosclerosis. *Circulation* 2018;137(25):2741-2756.

Hunt, G.J.*, et al.* dtangle: accurate and robust cell type deconvolution. *Bioinformatics* 2019;35(12):2093-2099.

Hyvärinen, A., Karhunen, J. and Oja, E. Independent Component Analysis Wiley-Interscience; 2001.

Kuhn, A.*, et al.* Cell population-specific expression analysis of human cerebellum. *BMC Genomics* 2012;13:610.

Kuhn, A.*, et al.* Population-specific expression analysis (PSEA) reveals molecular changes in diseased brain. *Nat Methods* 2011;8(11):945-947.

Li, C., Johnson, W.E. and Rabinovic, A. Adjusting batch effects in microarray expression data using empirical Bayes methods. *Biostatistics* 2006;8(1):118-127.

Newman, A.M.*, et al.* Robust enumeration of cell subsets from tissue expression profiles. *Nat Methods* 2015;12(5):453-457.

Parker, S.J.*, et al.* Identification of Putative Early Atherosclerosis Biomarkers by Unsupervised Deconvolution of Heterogeneous Vascular Proteomes. *J Proteome Res* 2020;19(7):2794-2806.

Sathe, A.*, et al.* Single-Cell Genomic Characterization Reveals the Cellular Reprogramming of the Gastric Tumor Microenvironment. *Clin Cancer Res* 2020;26(11):2640-2653.

Storey, J.D. and Tibshirani, R. Statistical significance for genomewide studies. *Proc Natl Acad Sci U S A* 2003;100(16):9440-9445.

Wang, J., Roeder, K. and Devlin, B. Bayesian estimation of cell type-specific gene expression with prior derived from single-cell data. *Genome Res* 2021.

Wang, N.*, et al.* Mathematical modelling of transcriptional heterogeneity identifies novel markers and subpopulations in complex tissues. *Scientific Reports* 2016;6:18909.

Wang, Y., Adali, T. and Lo, S.C. Automatic threshold selection using histogram quantization. *J Biomed Opt* 1997;2(2):211-217.

Wang, Y.*, et al.* Quantification and Segmentation of Brain Tissues from MR Images: A Probabilistic Neural Network Approach. *IEEE Trans Image Process* 1998;7(8):1165-1181.

Yates, J. and Boeva, V. Deciphering the etiology and role in oncogenic transformation of the CpG island methylator phenotype: a pan-cancer analysis. *Brief Bioinform* 2022.

Yu, G.*, et al.* Matched Gene Selection and Committee Classifier for Molecular Classification of Heterogeneous Diseases. *J. Mach. Learn. Res.* 2010;11:2141-2167.

Yu, G.*, et al.* PUGSVM: a caBIG analytical tool for multiclass gene selection and predictive classification. *Bioinformatics* 2011;27(5):736-738.

Zhong, Y.*, et al.* Digital sorting of complex tissues for cell type-specific gene expression profiles. *BMC Bioinformatics* 2013;14:89.
